# Postpartum ovarian vein thrombosis: a case report

**DOI:** 10.1097/MS9.0000000000000838

**Published:** 2023-05-12

**Authors:** Zeinab El Mawla, Bilal Damen

**Affiliations:** a Faculty of Medicine, Department of Pulmonary and Critical Care, Lebanese University; b Department of Pulmonary and Critical Care, Beirut Cardiac Institute, Beirut, Lebanon

**Keywords:** anticoagulation, case report, ovarian vein thrombosis, postpartum, pregnancy, thrombosis

## Abstract

Ovarian vein thrombosis (OVT) is a rare condition most frequently seen in the immediate postpartum period. Typical symptoms include pelvic pain, fever, and abdominal mass. Although a rare diagnosis, OVT can potentially cause fatal complications; thus, early recognition and prompt treatment is important.

**Case presentation:**

We present a case of a 30-year-old lady, G4P3A0, previously healthy, who presented at 29+3 weeks to the hospital for preterm premature rupture of membrane. The patient had a Cesarean section (C-section), which was complicated with uterine atony and massive bleeding controlled by emergent intrauterine balloon tamponade and uterine artery embolization. The next day, the patient complained of new-onset right lower quadrant abdominal pain, for which she had an abdominal and pelvic computed tomography (CT) scan that showed OVT. Thus, she was started on anticoagulants, and discharged home.

**Conclusion:**

OVT is a rare disorder that has been described as occurring mainly during the postpartum period, after pelvic surgery, or in women with gynecological malignancies. Clinical features of OVT include fever, abdominal pain and tenderness, and a palpable abdominal mass. Diagnosis can be obtained using CT, magnetic resonance (MR), or ultrasound (US) Doppler. Treatment includes a combination of anticoagulants and antibiotics. Mortality is low nowadays.

## Introduction

Highlights

It is a case report study.

- Ovarian vein thrombosis (OVT) is a rare condition most frequently seen in the immediate postpartum period.
- Typical symptoms include pelvic pain, fever, and abdominal mass.
- Diagnosis can be obtained using computed tomography (CT), magnetic resonance (MR), or ultrasound (US) Doppler.
- Treatment includes a combination of anticoagulants and antibiotics.
- Mortality is low nowadays.

Ovarian vein thrombosis (OVT) is a rare condition most frequently seen in the immediate postpartum period. OVT has been reported in ~2% of births by Cesarean section (C-section) and in 0.05–0.18% of vaginal births^1^. Typical symptoms include pelvic pain, fever, and abdominal mass^2^. OVT can be associated with gynecological malignancies, pelvic surgeries, pelvic inflammatory disease (PID), and hypercoagulable states^2^. The incidence of idiopathic OVT is exceedingly rare. Only a few cases with no known etiology have been reported to date in healthy patients. Although a rare diagnosis, OVT can potentially cause fatal complications; thus, early recognition and prompt treatment is important.

## Case presentation

A 30-year-old lady, G4P3A0, previously healthy, non-smoker, and non-alcohol user, presented at 29+3 weeks to the hospital for preterm premature rupture of membrane. The patient had early and regular prenatal care; she used to see her health care provider every 4 weeks and denied any history of complicated pregnancy or delivery. Her outpatient medications included prenatal vitamins and iron supplements. Upon presentation, the patient’s temperature was 37°C, heart rate was 89 beats per minute, blood pressure was 105/60 mmHg, respiratory rate was 24 breaths per minute, and oxygen saturation was 98% on room air oxygen. She was clinically and hemodynamically stable with regular heart sounds, clear lungs, soft abdomen, and no lower limb edema. Laboratory tests showed hemoglobin 9.7, hematocrit 30, and a white blood cell count of 10 000 with 90% neutrophils.

The patient was started on dexamethasone 6 mg intramuscular injection every 12 h in order to accelerate the maturation of the fetal lungs and rocephine 2 g intravenously once daily. On day 3, the patient had a C-section, which was complicated with uterine atony and massive bleeding; she thus received 4 l of lactate ringer solution, 4 units of packed red blood cells, 2 units of fresh frozen plasma, and 1 pool of platelets with tranexamic acid and calcium gluconate.

Hemostasis could not be achieved except after emergent intrauterine balloon tamponade and uterine artery embolization.

The patient was then transferred to the intensive care unit (ICU) for observation. Upon arrival in the ICU, the patient’s vitals were stable, with a blood pressure of 90/60 mmHg, pulse of 98, and SpO_2_ of 97% on room oxygen. The patient was somnolent but conscious and cooperative, oriented to time, place, and persons; pallor was noted with slight jugular venous distension, but otherwise clear lungs on auscultation. The uterine drain showed a small amount (50 cc) of serosanguinous fluid. Laboratory tests revealed hemoglobin 10, hematocrit 31, international normalized ratio (INR) 1.66, partial thromboplastin time (PTT) 39, D-dimer 185 052, and creatinine and electrolytes within the normal range.

**Figure 1:**
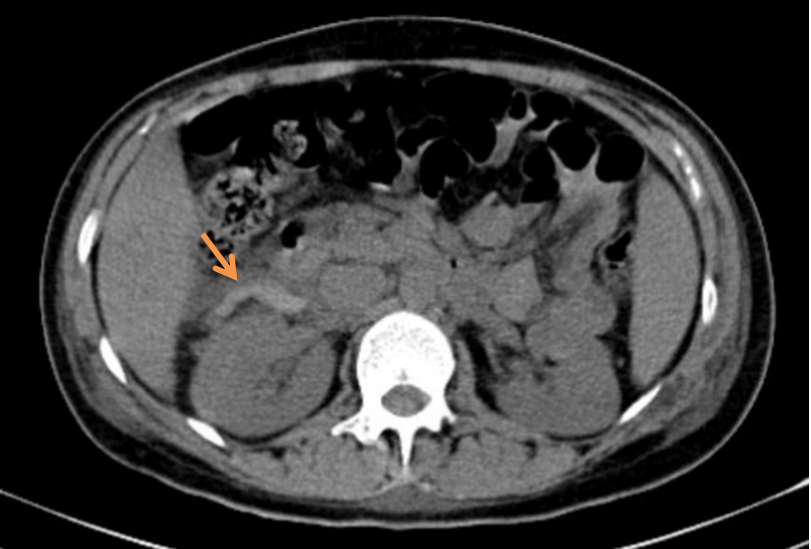
Computed tomography scan showed a tortuous right ovarian vein with surrounding amount of fluid suggestive of ovarian vein thrombosis.

**Figure 2:**
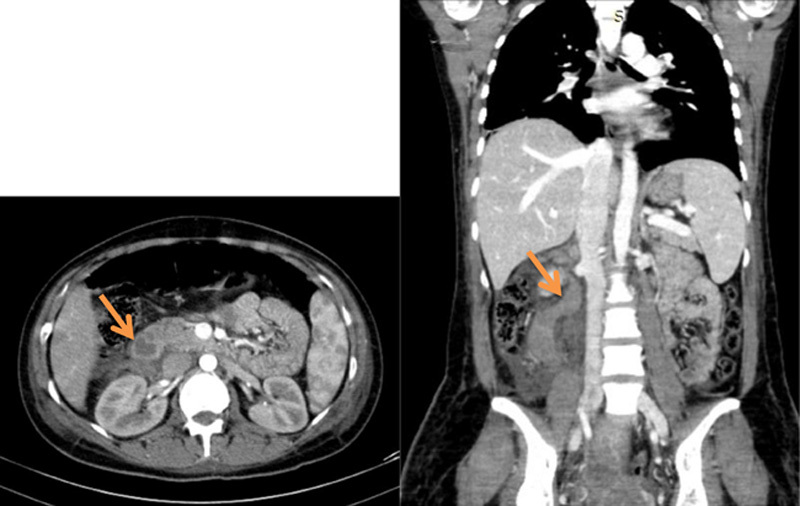
Computed tomography angiography with venous time showing right ovarian vein thrombosis.

On the next day of ICU stay, the patient complained of new-onset right lower quadrant abdominal pain for which she had an abdominal and pelvic computed tomography (CT) scan. The CT scan showed an enlarged uterus without hematoma, pelvic intraperitoneal drainage, and dilated and tortuous right ovarian vein with surrounding amount of fluid suggestive of OVT (Fig. 1). A complementary CT angiography with venous time was then performed and the diagnosis was confirmed (Fig. 2). The patient was started on heparin 5000 units intravenous bolus followed by 20 000 units of continuous heparin intravenous drip with PTT monitoring every 6 h and complete blood count monitoring every 12 h to check hemoglobin and to avoid the risk of rebleeding. Two days later, acenocoumarol was initiated, and bridging with heparin for three more days with therapeutic INR was achieved. The patient was discharged on acenocoumarol and advised for follow-up in 2 weeks.

## Discussion

OVT is a type of unusual-site venous thromboembolism (VTE) involving the female gonadal veins. OVT is a rare condition, with an incidence of around 60 times less common than lower limb deep vein thrombosis (DVT)^3^. However, it has been more frequently detected in recent years due to the widespread use of radiological imaging and its technical improvement^3^. OVT complicates ~0.01–0.18% of pregnancies 6–8 and is more common after a Cesarean delivery than a vaginal delivery^4^. Postpartum OVT occurs with a peak around 2–6 days after delivery or miscarriage/abortion, and in 90% of women, it arises within 10 days^4–6^. Pregnancy-related OVT is usually diagnosed in women in their early 30s^7,8^, while the other types of OVT involve older females with mean age ranging from 40 to 60 years in different studies^3,7–9^. The right ovarian vein is more frequently involved (70–80% of cases), due to the anatomical difference in venous drainage; occasionally, the thrombosis can be bilateral (around 10% of cases)^4,10^.

The most common risk factors for OVT are pregnancy, recent surgery, pelvic infections, estrogen-containing hormone treatment, and malignancy, although with variable prevalence in different studies. Malignancies associated with the development of OVT include mainly genitourinary cancers (such as uterine or ovarian tumors), followed by gastrointestinal cancers^3,8^. Finally, a percentage of 4–16% of OVT has no causative factor, and these forms are classified as idiopathic OVT^3,7^.

The most common symptom of OVT is lower quadrant abdominal pain, usually on the side of the thrombosed vein^7,11^. The pain may radiate to upper abdomen, the flank, or the groin^12^. The most typical finding, although reported in only 46% of cases^10^, is a palpable cord-like abdominal mass, extending from the ovary to the paracolic gutter and corresponding to the thrombosed ovarian vein^12^. Nonspecific symptoms such as nausea, vomiting, anorexia, malaise, and ileus are reported, but they are usually mild^7^. The presence of OVT has been evidenced in more than half of the women with puerperal pelvic thrombophlebitis. Asymptomatic OVT is frequent in patients with malignancies, in whom it might be an incidental finding on CT scans performed during follow-up visits^13^.

Diagnosis of OVT can be obtained using CT, magnetic resonance (MR), or ultrasound (US) Doppler. However, there is no definite consensus regarding the gold standard test, as only a few studies specifically evaluated sensitivity and specificity^13,14^. US Doppler is usually the first-line imaging, due to its safety, low cost, and wide availability. However, US is an operator-dependent imaging and can often be inconclusive due to obesity or the presence of abdominal meteorism, which interferes with the visualization of the ovarian veins^15,16^. CT scan (contrast-enhanced CT or CT venography) can better identify the presence and the extension of the thrombosis and is nowadays the reference imaging^15,16^. However, CT scanning involves the use of iodinated contrast medium and ionizing radiation, and contraindications include renal insufficiency, pregnancy, and previous contrast allergy. Specificity of CT has been reported to be 62–99% and sensitivity to be 77–100%^17^. MR has the benefit of not using iodinated contrast medium or ionizing radiation; however, disadvantages include higher costs, longer execution time, and non-availability for immediate use. Unenhanced MR can allow the direct visualization of the thrombus^15,16^. Specificity of MR has been reported to be roughly 100% and sensitivity ~92–100%; thus, MR is indicated when the suspicion of OVT persists and CT scan is inconclusive^15,16^.

Treatment of OVT includes a combination of anticoagulants and antibiotics. According to the guidelines of the Canadian Society of Obstetricians and Gynaecologists addressing pregnancy-related VTE, parenteral broad-spectrum antibiotics should be commenced as soon as the diagnosis of OVT is confirmed and administered for at least 48 h after fever resolution, considering longer duration in septicemic patients^18^. Furthermore, anticoagulation at a therapeutic dose for 1–3 months is suggested^18^. The guidelines of the British Committee for Standards in Haematology recommend an anticoagulant treatment duration of 3–6 months for women with postpartum OVT and suggest no treatment for incidentally detected isolated OVT in cancer patients^19^. Broad-spectrum antibiotics are crucial if there is a concomitant infection^17^. Anticoagulation follows the principles of the management of usual-site VTE. In the study by Assal *et al*.^8^, women with isolated OVT received parenteral anticoagulation in the acute phase, with either low-molecular-weight heparin (LMWH) or unfractionated heparin, and the majority of them were subsequently switched to vitamin K antagonists (VKAs). The novel direct oral anticoagulants (DOACs) were not specifically evaluated in patients with OVT, as unusual-site VTE was excluded from the large phase III randomized controlled trials evaluating their efficacy and safety in DVT and pulmonary embolism (PE)^20^. Anticoagulant treatment duration was variable in the different studies. Expert opinions suggest a definite anticoagulant treatment duration of 3 months for symptomatic postpartum OVT^17^. There are only a few studies reporting the risk of bleeding of OVT patients^3^. There are a few reports regarding the use of thrombolytic drugs (e.g. alteplase, urokinase)^21^. However, due to the associated high risk of bleeding, they should be reserved for selected cases of women with massive thrombosis. Retrievable inferior vena cava filters are indicated only when there are absolute contraindications to the anticoagulant treatment (e.g. active bleeding, undelayable surgery)^12^. Surgical treatments (e.g. ovarian vein excision or ligation) are rarely performed nowadays^22^.

Possible complications described in patients with OVT include thrombus progression into the left renal vein (for left OVT) or into the inferior vena cava (for right OVT) or PE, recurrent VTE, and pelvic congestion syndrome^3,8,9^. In approximately a quarter of patients, the thrombosis might have already extended into other veins at the time of diagnosis^7^. Rare complications of OVT include ovarian infarction with potential influence on fertility^1^, and obstruction of the right ureter, which can lead to hydronephrosis and renal failure^23^. The survival of both OVT and DVT women was decreased when compared with the general population^3^. Mortality due to OVT is less than 5% nowadays due to the use of antibiotics and anticoagulants^23^.

Our patient was diagnosed with a postpartum OVT. She was initially started on LMWH and later transitioned to oral warfarin therapy. The patient’s abdominal pain subsided soon after starting anticoagulation, and she was discharged with instructions to follow-up with hematology and a plan to repeat imaging in 3 months to determine the need for continuing anticoagulation.

The work has been reported in line with the SCARE 2020 criteria^24^.

## Conclusion

OVT is a rare disorder that has been described as occurring mainly during the postpartum period, after pelvic surgery, or in women with gynecological malignancies. Clinical features of OVT include fever, abdominal pain and tenderness, and a palpable abdominal mass, combined with nonspecific gastrointestinal symptoms. Since OVT is one of the causes of puerperal fever, it should be suspected in women with fever and pelvic abdominal pain within a week after delivery, especially if they had a Cesarean section. US Doppler is usually the first-line imaging, as it can be easily performed for the evaluation of puerperal fever or abdominal pain. However, MR or CT scan is suggested to confirm the presence and extension of OVT, because US can often be inconclusive. Due to a treatment combination of anticoagulation and parenteral broad-spectrum antibiotics, mortality is low nowadays. While no anticoagulation has been suggested for incidentally detected cancer-associated OVT, anticoagulant treatment duration of 3–6 months has been recommended for postpartum OVT. Evidence is lacking on the optimal management of other types of OVT.

## Ethical approval

Ethical approval was not applicable.

## Consent

Written informed consent was obtained from the patient’s family for the publication of this case report and any accompanying images. A copy of the written consent is available for review upon request by the Editor-in-Chief of this journal.

## Sources of funding

The authors declare that the research is not funded by any funding agency.

## Author contribution

Z.E.M. and B.D.: contributed to the management of the case and the conceptualization and planning of the case report; Z.M.: helped in the preparation of the manuscript. Both authors read and approved the final manuscript.

## Conflicts of interest disclosure

The authors declare that there are no conflicts of interest.

## Research registration unique identifying number (UIN)

Not applicable.

## Guarantor

Zeinab El Mawla.

## Data availability statement

No additional data are available.

## Provenance and peer review

Not commissioned, externally peer-reviewed.

## Acknowledgement

Not applicable.
